# Alternative crop residue management practices to mitigate the environmental and economic impacts of open burning of agricultural residues

**DOI:** 10.1038/s41598-024-65389-3

**Published:** 2024-06-22

**Authors:** Rutjaya Prateep Na Talang, Warangluck Na Sorn, Sucheela Polruang, Sanya Sirivithayapakorn

**Affiliations:** 1 Environmental Engineering Department, Faculty of Engineering, Environmental Modeling Consultant Center, Kasetsart University, Bangkok, 10900 Thailand; 2 Environmental Engineering Department, Faculty of Engineering, Kasetsart University, Bangkok, 10900 Thailand

**Keywords:** Biochar, Circular economy, PM_2.5_, Corn residue, Rice straw, Sugarcane leaves, Environmental sciences, Environmental impact

## Abstract

Deliberate open burning of crop residues emits greenhouse gases and toxic pollutants into the atmosphere. This study investigates the environmental impacts (global warming potential, GWP) and economic impacts (net cash flow) of nine agricultural residue management schemes, including open burning, fertilizer production, and biochar production for corn residue, rice straw, and sugarcane leaves. The environmental assessment shows that, except the open burning schemes, fossil fuel consumption is the main contributor of the GWP impact. The fertilizer and biochar schemes reduce the GWP impact including black carbon by 1.88–1.96 and 2.46–3.22 times compared to open burning. The biochar schemes have the lowest GWP (− 1833.19 to − 1473.21 kg CO_2_-eq/ton). The economic assessment outcomes reveal that the biochar schemes have the highest net cash flow (222.72—889.31 US$_2022_/ton or 1258.15–13409.16 US$_2022_/ha). The expenditures of open burning are practically zero, while the biochar schemes are the most costly to operate. The most preferable agricultural residue management type is the biochar production, given the lowest GWP impact and the highest net cash flow. To discourage open burning, the government should tailor the government assistance programs to the needs of the farmers and make the financial assistance more accessible.

## Introduction

Similarly to carbon emissions from livestock^1^, carbon emissions also arise from crop farming and the burning of crop residues^2^. In particular, the open burning of agricultural residues to clear cultivated fields of harvest by-products is widely practiced in many developing countries in Africa, South Asia, and Southeast Asia. Deliberate open burning of crop residues is a major contributing factor to local and transboundary haze and air pollution^3–5^. The impacts of open burning vary from region to region, depending on crop types, burning conditions, and seasons^6^.

The incomplete combustion of open burning of biomass emits greenhouse gases (GHG) and toxic pollutants into the atmosphere, including carbon monoxide (CO), carbon dioxide (CO_2_), methane (CH_4_), nitrogen oxides (NO_x_), sulfur oxides (SO_x_), volatile organic compounds (VOCs), and particulate matter (PM_2.5_ and PM_10_)^3,7,8^. Of particular concern are fine particles that are 2.5 microns or less in diameter (i.e., PM_2.5_). PM_2.5_ are so small that they can travel deeply into the respiratory tract and lodge inside the lungs, causing throat and lung irritation, lung cancer, and other respiratory diseases^9^.

Black carbon is a component of fine particulate matter (i.e., PM_2.5_) that has been linked to respiratory and cardiovascular diseases and even premature deaths. Black carbon also contributes to worsening climate change. According to Bond, et al.^6^, the global warming potential for a 100-year time horizon of black carbon is between 120 and 1800, with an average of 900. As a result, cutting down black carbon emissions slows down climate change, improves air quality, and reduces human health risks.

According to IQAir^10^, the air quality in northern Thailand is very poor, especially during the dry season when PM_2.5_ from local and transboundary illegal crop burning and forest fires are alarmingly high. The concentrations of PM_2.5_ in the northern region of the country routinely exceed (i.e., 400 percent higher) the World Health Organization standards for PM_2.5_^11^. In the province of *Chiangmai*, which is the cultural and economic hub of northern Thailand, there were only 43 days, during January–April 2021, with the average daily PM_2.5_ levels below 50 μg/m^3^, while the average daily PM_2.5_ levels exceeding 50 μg/m^3^ lasted for two consecutive months^12^. The worsening air quality coincided with the sheer number of reported hotspots (101,869 hotspots) caused by open burning of crop residues and forest fires between January–May 2021^13^. Compounded by agricultural expansion and the El Niño phenomenon, the PM_2.5_ situation would worsen if open burning is left unchecked^14,15^.

The current agricultural residue management strategies to curb open burning include building awareness of the impacts of open burning, promoting long-term crops, boycotting crops from farms that practice open burning, and outright banning of open burning. However, the existing strategies attach greater emphasis to punishment than reward. In other words, the current agricultural residue management strategies view farmers as part of the problem rather than the solution. As the demand for short-term commercial crops, e.g., rice, sugarcane, and corn, continually increases^16^, switching from open burning to zero burning using land clearing equipment is an environment friendly ideal solution to tackle open burning pollution.

The zero burning practices, e.g., conversion of crop residues into fertilizers and biochar products, are compatible with the concept of circular economy^17–19^. However, the switching from open burning to zero burning involves large capital investment and operation and maintenance costs. Such financial burdens could act as a deterrent to shifting from open burning to zero burning practices.

The focus of this current research is the environmental and economic impacts of three types of agricultural residue management of three different crop residues. The types of agricultural residue management being studied include open burning (OB), fertilizer production (Fe), and biochar production (BC); and the three crop residues are corn residue (C), rice straw (R), and sugarcane leaves (S). This study aims to perform comparative assessment of environmental (the global warming potential (GWP)) and economic impacts (net cash flow) of the nine agricultural residue management schemes and the most preferable type of crop residue management is selected. The nine agricultural residue management schemes include C-OB, C-Fe, C-BC, R-OB, R-Fe, R-BC, S-OB, S-Fe, and S-BC. Undertaking this work is crucial for the international community to attain environmental sustainability, food security, economic development, public health, and fostering international cooperation. The international community, including governmental bodies and organizations like the UN and the World Bank, can utilize the findings on environmental and economic impacts to determine the optimal approach to managing crop residue.

In this research, revenues from avoidance carbon emission credits are included for the Fe and BC options. This is comparable to the carbon credit projects to reduce emissions from non-energy open biomass burning of forests^20^ and those to reduce emissions from the household biomass burning for energy, i.e., the Cookstove Project^21^. Specifically, in the assessment, the carbon credits are comprised of: (i) the avoided GHG emissions after switching from open burning to zero burning (i.e., fertilizer and biochar production) and (ii) the carbon storage from the conversion of crop residues to biochar.

## Materials and methods

**Figure 1:**
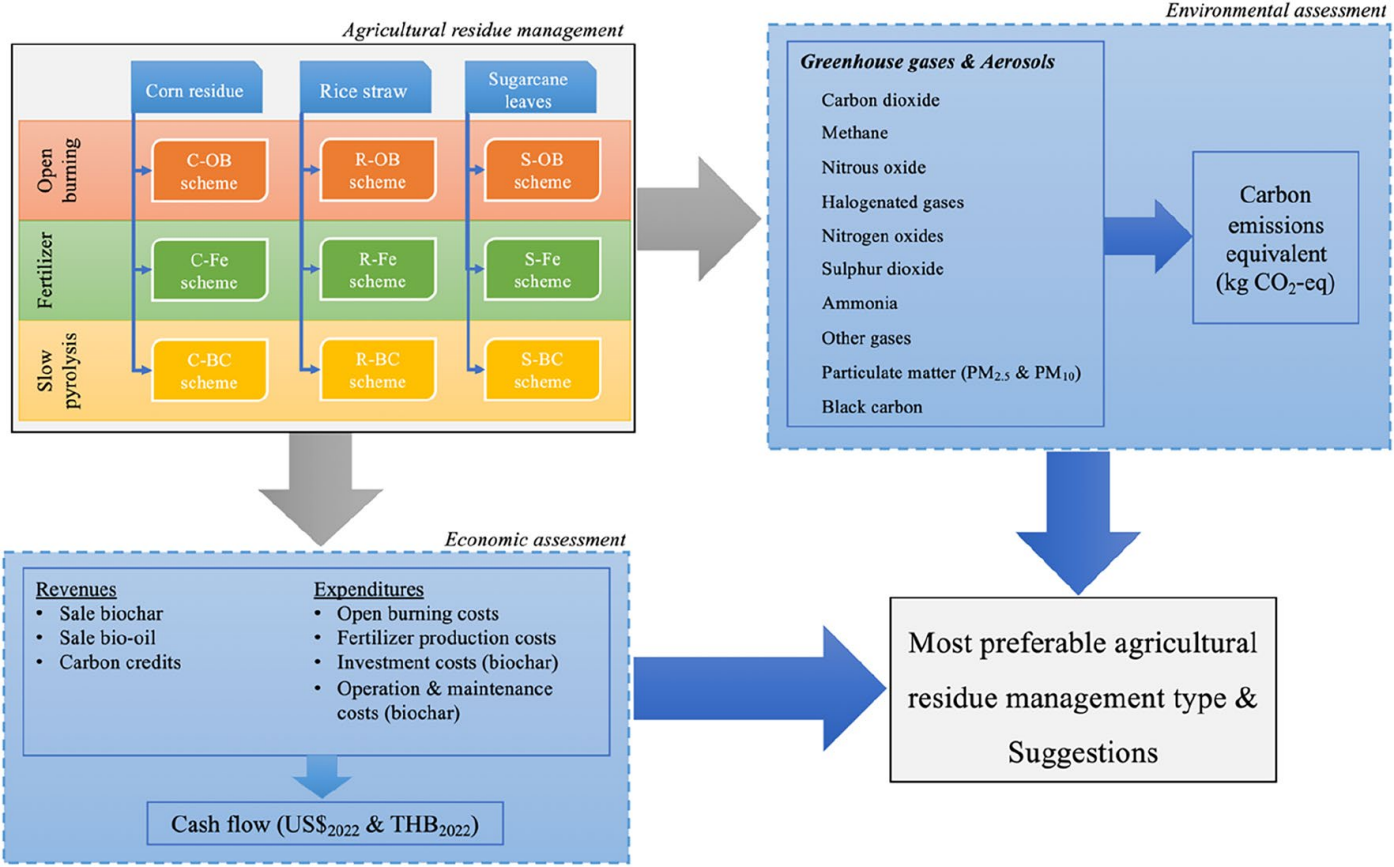
The methodology framework of this research.

The secondary data (i.e., the emission factors, the inventory data and the economic data) of the three crop residues (i.e., corn residue, rice straw, and sugarcane leaves) that are used for this study, are gathered from existing peer-reviewed publications and reports by local government agencies and international organizations. Figure 1 shows the methodology framework of this research. The study focuses on three types of agricultural residue management (i.e., open burning, fertilizer production, and biochar production using slow pyrolysis) treating three different crop residues (i.e., corn residue including corn cobs, leaves, and stalks; rice straw; and sugarcane leaves).

Essentially, this research comparatively assesses the environmental and economic impacts of nine agricultural residue management schemes and the most preferable type of agricultural residue management generates the lowest GWP impact and the highest net cash flow.

In the study, the GWP is calculated based on the inventory data of GHG emissions^6,22^. Unlike the GWP calculation by the Intergovernmental Panel on Climate Change (IPCC), the GWP of open burning of this study includes black carbon emissions.

### Agricultural residue management schemes

In this study, the emission factors of the pollutants from open burning between 1998 and 2021 are obtained from the existing researches^3–5,7,8,22–41^ and provided in Table 1. This study uses the average emission factors of air pollutants of the three crop residues (i.e., corn residue, rice straw, and sugarcane leaves) to assess the GWP impact. In practice, sugarcane farmers burn sugarcane crops before harvest and the crops are harvested manually, whereas corn and rice farmers burn the agricultural residues after harvest.

**Table 1:** The average emission factors of pollutants from open burning.

Emission factors (g/kg)	Corn residue	Rice straw	Sugarcane leaves
PM_2.5_	9.11^3,4,7,30,32^	12.34^42^	10.36^38–40^
PM_10_	14.86^32^	7.84^42,43^	No data
CO	64.66^4,7,26–28,30,31,34^	68.22^4,5,7,8,26,27,34,36,42–44^	63.14^38–41^
CO_2_	1663.14^4,7,26,27,30,34^	1229.50^4,5,7,8,26,27,34,36,42,44^	1379.50^38–41^
CH_4_	4.40^30^	3.52*^22–25^	3.52*^22–25^
N_2_O	0.14^30^	0.07*^22–25^	0.07*^22–25^
NO_x_	3.42^7,26–28,30,34^	2.68^7,26,27,34,36^	1.50^38^
NH_3_	0.66^7,28,30^	1.54^7,8,44^	1.50*^22–25^
SO_2_	0.35^7,26–28,30^	0.26^7,8,26,27,36,43^	0.40*^22–25^
Black carbon	1.24^7,27,28,30,32,33^	1.13^7,27,33,35,45^	0.61^37^

*indicates that the values are the average of the emission factors of all agricultural residues due to unavailability of data specific to the particular crops.

Apart from open burning, agricultural residues could also be converted into organic fertilizers. The common practice is to chop and blend it with the soil to enrich the agricultural fields. The conversion of agricultural waste into fertilizers helps mitigate emissions from open burning. In this research, the GWP calculation of the fertilizer production schemes excludes emissions from the natural decomposition of agricultural residues.

The slow pyrolysis of biochar is carried out in an oxygen limited environment at 350 – 500 ºC, with typical heating rates between 1 and 30 °C min^−1^^46^ to improve the quality and yield of biochar^47^. In this research, the biochar yields of agricultural residues are obtained from Tomczyk, et al. ^48^.

### Environmental assessment

The GWP assessment of the nine agricultural residue management schemes adheres to the life cycle assessment (LCA) methodology, encompassing four steps: (i) defining system boundaries, functional units, and assumptions; (ii) analyzing inventory data; (iii) conducting life cycle impact assessment (LCIA); and (iv) analyzing outcomes to pinpoint the agricultural residue management scheme with the least environmental impact^49^.

(i) Defining system boundaries: The system boundary for the nine agricultural residue management schemes is gate-to-gate life cycle, covering various management types (e.g., open burning, fertilizer production, biochar production) and agricultural residue transportation. In this study, transportation applies to biochar schemes, where crop residues are transported within a 100 km radius from fields to production facilities via trucks. The functional unit is set at 1000 kg of agricultural residues.
(ii) Analyzing inventory data: Inventory data for agricultural residue management in Thailand from 2001 to 2023 are sourced from multiple references^16,50–58^. Biochar production data are obtained from studies by Tomczyk, et al.^48^,Sahoo, et al.^59^. Input and output data for the three types of agricultural residue management are presented in Tables 2, 3, and 4. This study employs.
(iii) Conducting life cycle impact assessment (LCIA The GWP of the nine agricultural residue management schemes is assessed using IPCC 2021 methodology in openLCA 2.0 software, with LCIA conducted via the ecoinvent database. To incorporate black carbon emissions from incomplete combustion, which are not covered by the IPCC method, the open burning schemes (C-OB, R-OB, S-OB) include the average GWP of 900 for black carbon emissions (100-year time horizon)^6^ in their calculations. Conversely, the fertilizer (C-Fe, R-Fe, S-Fe) and biochar schemes (C-BC, R-BC, S-BC) reflect negative emissions due to the absence of pollutants emitted from open burning, including N_2_O, CH_4_, and black carbon.

**Table 2:** The inventory data of open burning of agricultural residues.

Inventory data	Unit	Open burning		
		Corn residue	Rice straw	Sugarcane leaves
Input				
Corn residue	kg/ha	9242.35		
Rice straw	kg/ha		2530.28	
Sugarcane leaves	kg/ha			15,078.11
Output				
Ash	kg/ha	184.85	101.21	1362.14
*Emissions to air*				
PM_2.5_	kg/ha	84.16	31.24	156.26
PM_10_	kg/ha	137.34	19.84	No data
CO	kg/ha	597.61	172.63	952.06
CO_2_	kg/ha	15,371.32	3110.97	20,800.25
CH_4_	kg/ha	40.67	53.13	53.13
N_2_O	kg/ha	1.29	1.06	1.06
NO_x_	kg/ha	31.61	6.78	22.62
NH_3_	kg/ha	6.13	3.89	22.61
SO_2_	kg/ha	3.19	0.67	6.03
Black carbon	kg/ha	11.48	2.86	9.20

Office of Agricultural Economics^16^,Office of the Cane and Sugar Board^50^,Silalertruksa and Gheewala^51^,Silalertruksa, et al.^52^,Supasri, et al.^53^,Thailand Environment Foundation^54^,Towprayoon, et al.^55^,Zhang, et al.^56^.

**Table 3:** The inventory data of fertilizer production.

Inventory	Unit	Fertilizer production		
		Corn residue	Rice straw	Sugarcane leaves
Input				
Biomass	kg/ha	9242.35	2530.28	15078.11
Diesel—chopping biomass	L/ha	284.91	78.00	75.00
Diesel—blending biomass with soil	L/ha	9.38	16.00	10.44
Output				
Fertilizer	kg/ha	9242.35	2530.28	15078.11

Silalertruksa and Gheewala^51^,Supasri, et al.^53^,Thailand Environment Foundation^54^,Chaneeparp^57^,Phettharawadee^58^.

**Table 4:** The inventory data of biochar production.

Inventory	Unit	Biochar production		
		Corn residue	Rice straw	Sugarcane leaves
Input				
Biomass	kg dry	2695.42	2985.07	2715.92
Biochar yield (biochar/biomass)	%	37.10%	33.50%	36.82%
Electricity	kWh	234	234	234
Propane	L	1727	1727	1727
Transport of biomass	tkm	269.54	298.51	271.59
Output				
Biochar	kg	1000	1000	1000
Bio-oil (co-product)	kg	857.14	857.14	857.14
Emissions to air				
PM_2.5_	kg	0.02	0.02	0.02
PM_10_	kg	2.38	2.64	2.40
CO	kg	1.20	1.33	1.21
CO_2_	kg	4353.10	4820.90	4386.20
CH_4_	kg	0.26	0.29	0.27
NO_x_	kg	3.39	3.76	3.42
SO_2_	kg	0.06	0.07	0.06
Fixed carbon	%	72.30%	81.55%	78.59%

Tomczyk, et al.^48^,Sahoo, et al.^59^,Kumar, et al.^60^,Yaashikaa, et al.^61^,Wang, et al.^62^.

The carbon storage of biochar schemes is calculated by Eq. (1) based on the molecular weights of carbon (12 g/mole) and CO_2_ (44 g/mole), the fixed carbon percentage, and the biochar yield of agricultural residues.

1 $$\text{Carbon storage }\left(\text{kg }{\text{CO}}_{2}\right)=\frac{44}{12}\times \text{Fixed carbon percentage }\left(\text{\%}\right)\times \text{Biochar yield }(\text{kg})$$

In this study, carbon storage refers to the capturing of CO_2_ emissions and storing them in solid form (i.e., biochar), thereby preventing CO_2_ emissions from having an effect on climate. The carbon storage is thus regarded as negative emissions when incorporating into the GWP assessment.

The ISO14044:2006 standard, which covers LCA studies and life cycle inventory studies, requires that sensitivity analysis be performed to assess the reliability of the final outcomes^49^. According to IPCC^63^, fossil fuel consumption is the major contributor of GWP impact. However, modern diesel engines are more fuel-efficient and emit less GHG^64^. In the sensitivity, the diesel fuel consumption of the fertilizer schemes (C-Fe, R-Fe, S-Fe) is thus assumed to decrease by 10%, 20%, and 30%. Meanwhile, the distance between the harvested fields and the biochar production facilities of the biochar schemes (C-BC, R-BC, S-BC) are assumed to increase by 200, 300, and 500 km.

Analyzing outcomes: the agricultural residue management scheme with the least environmental impact is determined based on its lowest GWP.

### Economic assessment

In the economic assessment, agricultural residue managements excluding biochar production, occur at harvested fields, while for biochar production, crop residues are sold to production facilities, necessitating transportation. Revenue sources under the nine management schemes include biochar sales, bio-oil sales as an alternative to heavy fuel oil, and carbon credits from avoided emissions and carbon storage resulting from no open burning practices i.e., fertilizer production and biochar production.

In Table 4 (the inventory data of biochar production), the yields of biochar and bio-oil are 394.04–436.39 kg per ton of agricultural residues and 239.29–256.00 L per ton of agricultural residues, respectively^59,61^. The market prices of biochar and bio-oil are 9.38 US$ per kg (109.9 THB per kg) and 1.11 US$ per L (13 THB per L)^65^. Thailand’s average carbon credit between 2021 and 2023 is 6.40 US$ per ton CO_2_ (75.05 THB per ton CO_2_)^66^. In this study, the carbon credits consist of the avoided emissions from fertilizer and biochar production and the carbon storage in biochar. (Note: THB denotes the currency of Thailand or Thai baht.)

The expenditures (i.e., cash outflow) are obtained from Silalertruksa and Gheewala^51^,Thailand Environment Foundation^54^,Sahoo, et al.^59^,Energy Policy and Planning office^65^,Ministry of Labour^67^ and government reports between 2014 and 2023, including investment cost and operation and maintenance cost. For the fertilizer schemes (C-Fe, R-Fe, S-Fe), the major cost items (i.e., operation costs) include diesel fuels (2.94 US$/L or 34.45 THB/L) and wages (29.09 US$/day or 340.89 THB/day on average)^65,67^. Since the machines and equipment are rented (instead of purchase), the investment and maintenance costs are excluded for the fertilizer schemes. For the biochar schemes (C-BC, R-BC, S-BC), the investment cost in biochar production machinery and the maintenance cost are obtained from Sahoo, et al.^59^. The operation costs of the biochar schemes include feedstock, propane, electricity, diesel fuel for transportation, packaging, and labor.

The revenues and expenditures are converted into the year 2022 United States currency (US$) and Thai currency (THB) based on the purchasing power parity (PPP) and gross domestic product (GDP) deflator index to reconcile differences between US$ and THB^68^. Both currencies conversion equations are provided in Eq. (2) and (3), and the conversion factors of PPP and GDP deflator are tabulated in Table 5.

2 $$US\$_{2019} = \frac{{THB_{2019} \times PPP_{US\$ 2019} }}{{PPP_{THB2019} }}\;or\;THB_{2019} = \frac{{US\$_{2019} \times PPP_{THB2019} }}{{PPP_{US\$ 2019} }}$$

where THB_2019_ and US$_2019_ are the 2019 values of Thai baht (THB) and United States dollar (US$), and PPP_THB2019_ and PPP_US$2019_ are the exchange rate-adjusted purchasing power parity for Thai baht (THB) and United States dollar (US$).

3 $$US\$_{2022} = \frac{{US\$_{2019} \times D_{US\$ 2022} }}{{D_{US\$ 2019} }}\;or\;THB_{2022} = \frac{{THB_{2019} \times D_{THB2022} }}{{D_{THB2019} }}$$

where US$_2019_ and US$_2022_ are the 2019 and 2022 United States dollar (US$) values, THB_2019_ and THB_2022_ are the 2019 and 2022 Thai baht (THB) values, D_US$2019_ and D _US$2022_ are the 2019 and 2022 GDP deflator indexes of United States dollar (US$) and D_THB2019_ and D _THB2022_ are the 2019 and 2022 GDP deflator indexes of Thai baht (THB).

**Table 5:** The conversion factors for the economic assessment.

Item	Conversion factor
PPPUS$_2019_	1
PPPUS$_2022_	1
PPP_Thai2019_	12.6249
PPP_Thai2022_	11.7181
GDP_US$2019_	107.2859
GDP_US$2022_	121.5247
GDP_Thai2019_	154.6724
GDP_Thai2022_	162.6152

World Bank^68^.

### The most preferable type of agricultural residue management

The nine agricultural residue management schemes are assessed to determine the most environmentally and financially viable type of crop residue treatment for corn residue, rice straw, and sugarcane leaves. The selection criteria for the most environmentally and financially viable schemes or type are the lowest GWP impact and the highest net cash flow.

## Results and discussion

### Environmental assessment outcomes

The environmental LCA results indicate that, except the open burning schemes (C-OB, R-OB, S-OB), fossil fuel consumption is the main contributor to the GWP impact. The main contributor of the GWP impact for the fertilizer schemes (C-Fe, R-Fe, and S-Fe) is diesel fuel consumption, and that of the biochar schemes (C-BC, R-BC, and S-BC) is propane consumption.

**Figure 2:**
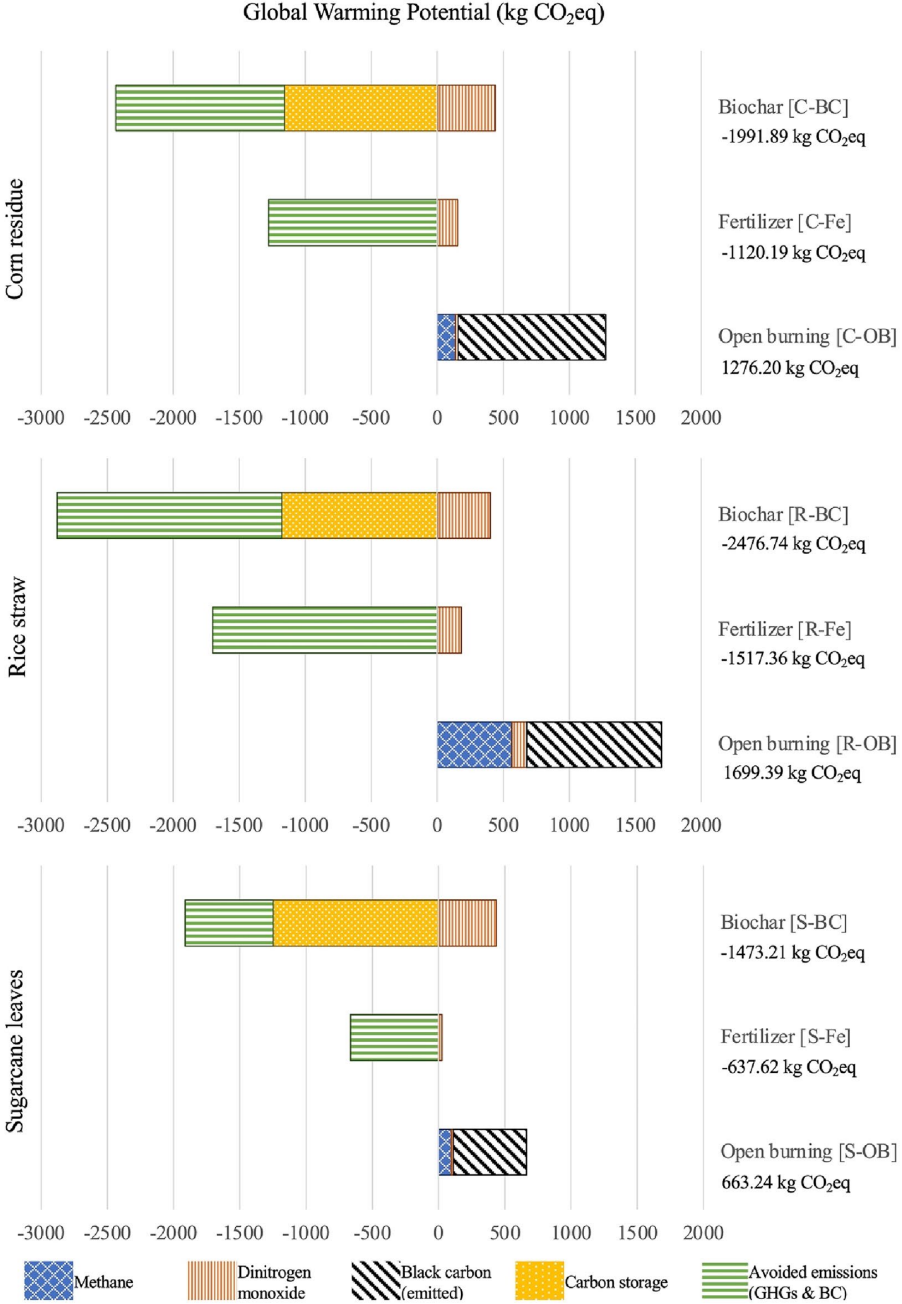
The global warming potential impact of the nine agricultural residue management schemes.

Overall, the fertilizer and biochar schemes reduce the GWP impact by 1.88 to 1.96 times and 2.46 to 3.22 times, respectively, compared to open burning schemes. Figure 2 shows the GWP impacts of the nine agricultural residue management schemes. The GWP impact of the biochar schemes are the lowest (− 1991.89 kg CO_2_-eq/ton corn residue, − 2476.74 kg CO_2_-eq/ton rice straw, and − 1473.21 kg CO_2_-eq/ton sugarcane leaves). The GWP impact of black carbon from corn residue is the highest (1117.50 kg CO_2_-eq/ton), followed by rice straw (1018.62 kg CO_2_-eq/ton) and sugarcane leaves (549.00 kg CO_2_-eq/ton) (Tables 1 and 2).

The avoided emissions of rice straw are the lowest (− 1699.39 kg CO_2_-eq/ton) for both fertilizer and the biochar schemes. In the biochar production, the carbon storage of sugarcane leaves (− 1248.02 kg CO_2_-eq/ton or − 18,817.74 kg CO_2_-eq/ha) was the highest, followed by corn residue (− 1156.86 kg CO_2_-eq/ton or − 10,692.12 kg CO_2_-eq/ha) and rice straw (− 1178.25 kg CO_2_-eq/ton or − 2981.30 kg CO_2_-eq/ha). The highest carbon storage of sugarcane leaves could be attributed to the higher percentage of fixed carbon, the higher biochar yield, and the highest agricultural residues per ha (Table 2).

The highest GWP of the open burning schemes could be attributed to air pollutants, especially black carbon, emitted during the burning of agricultural residues (i.e., corn residue, rice straw, and sugarcane leaves). Black carbon can lodge into the respiratory tract and inside the lungs, causing throat and lung irritation, lung cancer, and other respiratory diseases^9,69^.

Roberts, et al.^70^ studied the LCA of biochar production from corn residue and reported that the GWP impact of biochar production from corn residue is − 864 kg CO_2_-eq/ton, vis-a-vis the result of this research (− 715.69 kg CO_2_-eq/ton corn residue without avoided emissions). The higher GWP of biochar production of this study could be attributed to the farther distance between the harvested fields and the biochar production facilities (i.e., within a 100 km radius vs 15 km on average). Biochar is primarily utilized for carbon storage, and the carbon storage capacity of biochar depends on feedstock and pyrolysis temperature^48^.

The sensitivity analysis results reveal that the lower diesel fuel consumption (by 10%, 20%, and 30%) for the fertilizer schemes (C-Fe, R-Fe, S-Fe) and the farther distance between the harvested fields and the biochar production facilities for the biochar schemes (C-BC, R-BC, S-BC) have negligible effects on the GWP.

### Economic assessment outcomes

Table 6 presents the cash flow of the nine agricultural residue management schemes. The net cash flow (i.e., cash inflow—cash outflow) of the biochar schemes (C-BC, R-BC, and S-BC) are highest (222.72 US$_2022_/ton corn residue (2609.80 THB_2022_/ton corn residue) or 2058.42 US$_2022_/ha (24,120.67 THB_2022_/ha); 497.24 US$_2022_/ton rice straw (5826.65 THB_2022_/ton rice straw) or 1258.15 US$_2022_/ha (14,743.04 THB_2022_/ha); and 889.31 US$_2022_/ton sugarcane leaves (10,421.02 THB_2022_/ton sugarcane leaves) or 13,409.16 US$_2022_/ha (157,129.28 THB_2022_/ha)).

**Table 6:** The cash flow of the nine agricultural residue management schemes.

List		Corn residue			Rice straw			Sugarcane leaves		
		Open burning (C-OB)	Fertilizer (C-Fe)	Biochar (C-BC)	Open burning (R-OB)	Fertilizer (R-Fe)	Biochar (R-BC)	Open burning (S-OB)	Fertilizer (S-Fe)	Biochar (S-BC)
Cash inflow	Sale biochar			4092.74			3695.60			4061.85
	Sale bio-oil			293.99			265.46			291.77
	Carbon credits		7.17	12.76		9.72	15.86		4.08	9.44
	Carbon credits–process emissions		− 1.00	-2.83		− 1.17	− 2.57		− 0.16	− 2.81
	Carbon credits–avoided emissions		8.17	8.17		10.88	10.88		4.25	4.25
	Carbon credits–biochar as carbon storage			7.41			7.55			7.99
Total Revenues		0.00	7.17	4399.49	0.00	9.72	3976.93	0.00	4.08	4363.06
Cash outflow	Fertilizer		96.76			116.71			19.17	
	Labor–burning biomass	Practically 0			Practically 0			Practically 0		
	Labor–chopping biomass		1.51			1.51			1.51	
	Labor–mixing biomass		1.64			5.99			1.00	
	Diesel–burning biomass	Practically 0			Practically 0			Practically 0		
	Diesel–chopping biomass		90.63			90.63			14.62	
	Diesel–mixing biomass		2.98			18.59			2.04	
	Biochar			4176.77			3479.69			3473.75
	Investment cost			376.00			339.51			373.16
	Operation cost			3730.84			3077.03			3031.18
	Maintenance cost			69.93			63.14			69.40
Total Expenditures		Practically 0	96.76	4176.77	Practically 0	116.71	3479.69	Practically 0	19.17	3473.75
Net cash flow (Revenues—Expenditures)	US$_2022_/ton	0.00	− 89.58	222.72	0.00	− 107.00	497.24	0.00	− 15.09	889.31
	US$_2022_/ha	0.00	− 827.95	2058.42	0.00	− 270.73	1258.15	0.00	− 227.48	13409.16

Unit: US$_2022_ per ton agricultural residues.

More specifically, the cash inflow of the biochar production from corn residue is the highest (4399.49 US$_2022_/ton or 51,553.44 THB_2022_/ton), followed by sugarcane leaves (3663.06 US$_2022_/ton or 51,126.56 THB_2022_/ton) and rice straw (46,601.85 US$_2022_/ton or 46,601.85 THB_2022_/ton). The sale of biochar accounts for the largest proportion of cash inflow (3695.60–4092.74 US$_2022_/ton or 43,305.25–47,958.95 THB_2022_/ton), followed by the sale of bio-oil and the carbon credits.

The carbon credits of the fertilizer schemes are between 4.08–9.72 US$_2022_/ton (47.85 – 113.88 THB_2022_/ton) or 24.59–66.31 US$_2022_/ha (288.14–777.01 THB_2022_/ha), while those of the biochar schemes are between 9.44–15.86 US$_2022_/ton (110.56–185.88 THB_2022_/ton) or 40.14–142.27 US$_2022_/ha (470.33–1667.11 THB_2022_/ha), given that the average price of carbon credits in Thailand between 2021 and 2023 is 6.40 US$ ton CO_2_ (75.05 THB/ton CO_2_). The price of carbon credits in Thailand is substantially lower than the international prices of 30–120 US$ or 351.54–1406.17 THB_2022_^66,71^. The lower price of carbon credits in Thailand is partly attributable to the voluntary nature of the carbon markets in the country, as opposed to the mandatory market in many developed countries such as EU Emissions Trading System^72^. The study results also indicate that, in order for the farmers to switch from open burning to fertilizer production, the minimum prices of carbon credits from avoided emissions should be 30.07 US$/ton CO_2_ (352.31 THB/ton CO_2_), 76.92 US$/ton CO_2_ (901.34 THB/ton CO_2_) and 86.38 US$/ton CO_2_ (1012.15 THB/ton CO_2_) for sugarcane leaves, rice straw and corn residue, respectively.

The expenditures (i.e., cash outflow) of the biochar schemes are the highest (4176.77 US$_2022_/ton or 48,943.64 THB_2022_/ton for C-BC, 3479.69 US$_2022_/ton or 40,775.19 THB_2022_/ton for R-BC, and 3473.75 US$_2022_/ton or 40,705.54 THB_2022_/ton for S-BC). Meanwhile, the expenditures of the open burning schemes are practically zero.

In the existing researches, the economic benefits of biochar include a reduction in the use of chemical fertilizers and an improvement in crop yields^73–75^. Jeffery, et al.^73^ and Wang, et al.^74^ report that applying biochar at rates between 50 and 150 ton/ha in tropical soil can be effective. Specifically, Tisserant and Cherubini^75^ report a potential reduction in fertilizer use by 7% with biochar application. In our study, utilizing biochar could reduce the fertilizer usage (15-15-15 compound fertilizer) to 186.15 kg/ha for corn and rice, and 453.38 kg/ha for sugarcane, resulting in reduced fertilizer costs of 28.26 US$_2022_/ha (331.21 THB_2022_/ha) for both corn and rice, and 68.84 US$_2022_/ha (806.68 THB_2022_/ha) for sugarcane. Additionally, biochar has been found to increase crop yields by 10% to 42%^76^, with an average increase of 25%^73^. In this study, the improved crop yields of corn, rice, and sugarcane could result in increased cash inflows of 871.89, 1690.53 and 1426.76 US$_2022_/ha (10216.89, 19809.71 and 16754.06 THB_2022_/ha), respectively.

### The most preferable type of agricultural residue management

The environmental and economic assessment outcomes show that the conversion of crop residues to biochar (C-BC, R-BC, and S-BC) is the most preferable type of agricultural waste treatment because of the lowest GWP impact and the highest net cash flow. More specifically, the R-BC scheme has the lowest the GWP impact (− 2476.74 kg CO_2_-eq/ton), while the S-BC scheme has the highest net cash flow (889.31 US$_2022_/ton (10421.02 THB_2022_/ton) or 13409.16 US$_2022_/ha (157129.28 THB_2022_/ha)).

In 2019, the agriculture sector in Thailand emitted 56,766.32 Gg CO_2_-eq, accounting for 15.23% of total national GHG emissions^77^ excluded the black carbon emission. If this practice were adopted on a larger scale, assuming that 20% of each agricultural residues (corn residue, rice straw, and sugarcane leaves) are diverted from open burning to biochar production, GHG emissions would decreased by 17,345.99 Gg CO_2_-eq, representing 30.56% decrease in total agricultural GHG emissions, with black carbon emissions reduced by12641.24 Gg CO_2_-eq. Additionally, Thailand’s nationally determined contribution aims to unconditionally reduce GHG emissions by 30% from the business-as-usual scenario by 2030^77^. Switching 20% of the three agricultural residues to biochar could reduce GHG emissions by 4.69% compared to Thailand's business-as-usual emissions level in 2030^78^.

Given the practically zero cost of open burning and lax law enforcement, farmers in many developing countries, including Thailand, burn their crop residues to clear harvested fields off harvest by-products. However, smoke from deliberate burning of agricultural waste contains toxic gases and pollutants harmful to human health and the environment. To discourage open burning and promote the adoption of zero burning practices, the government needs to play the role of a facilitator rather than a police officer.

More specifically, the state authorities need to tailor the government assistance programs to the requirements of farmers and make them more accessible to the farmers, as opposed to imposing harsher punishments, such as imposing hefty fines or longer prison terms, which prove ineffective. The government could provide medium-term (5 years) zero-interest loans to farmers who plan to switch to zero burning. Since the loans carry zero interest rate and would not have to be repaid for the first two or three years, the farmers would be incentivized to abandon open burning and adopt the zero burning practices. In addition, amendments are required to existing laws and regulations so that land clearing machines and biochar equipment are subject to a zero rate of import customs duty. This adjustment would render the prices of these machines and equipment more affordable for farmers. These policies have the potential to decrease GWP impact by 633.24–1699.39 kg CO_2_-eq/ton of crop residues by promoting compliance and providing financial incentives. Choosing the biochar management method could result in farmers earning at least 222.72 US$ (2609.80 THB) per ton of crop residues or 1258.15 US$ (14743.04 THB) per hectare. This combination of measures and benefits could significantly sway farmers away from practicing open burning. Besides, the government should educate farmers about carbon credits and provide them with consultation and assistance to take part in the carbon projects as another source of income for farmers.

In the domain of incentivized carbon credit projects, various stakeholders collaborate to ensure their success and effectiveness. Government agencies play a pivotal role in this framework by making crucial decisions and establishing regulatory structures. They should prioritize tasks such as incorporating black carbon into greenhouse gas inventories for its specific contribution to both climate change and PM2.5, establishing certified parties and trading mechanisms, and introducing carbon pricing on agricultural products. To encourage participation, government agencies can set a legal framework for companies and businesses, such as setting carbon emission targets, to engage in carbon credit trading. Concurrently, they oversee the creation of carbon projects aimed at preventing further deforestation and the conversion of forests into agricultural land, while also encouraging participation by setting carbon emission targets. Politicians also play a significant role in this process as policy makers. They contribute to the development and enactment of legislation and regulations related to carbon emissions and offset projects. Through their decisions and actions, politicians shape the overall policy landscape and provide the necessary framework for the implementation of carbon credit initiatives.

Farmers, as key players, actively engage in sustainable practices and carbon markets. By refraining from open burning and selling carbon credits earned through sustainable farming methods, they not only generate additional income but also foster eco-friendly practices. Collaboration with various stakeholders, including government agencies and non-governmental organizations, allows farmers to access resources, technical support, and funding for implementing carbon projects and embracing sustainability.

Communities residing in regions with carbon offset projects reap benefits like job opportunities, infrastructure enhancement, and improved environmental quality. Their involvement is vital for project success and ensures equitable distribution of benefits. Furthermore, private sector entities, including companies and businesses, contribute by reducing carbon emissions and investing in carbon offset projects. They also engage in carbon trading to meet regulatory requirements and corporate sustainability goals.

Financial institutions play a significant role by offering funding and investment opportunities for carbon offset projects. They evaluate project feasibility, manage financial risks, and provide financial instruments such as carbon funds or green bonds to facilitate project advancement. Additionally, non-governmental organizations focused on environmental conservation assist in advocating for sustainable practices and facilitating community engagement. They serve as intermediaries, connecting project developers with funding sources or buyers for carbon credits.

Overall, the collaborative efforts of these stakeholders, including project developers and carbon standards bodies, ensure the successful implementation and integrity of incentivized carbon credit projects, fostering environmental stewardship and sustainable development.

## Conclusion

This research comparatively assesses the environmental and economic impacts of nine agricultural residue management schemes, including C-OB, C-Fe, C-BC; R-OB, R-Fe, R-BC; S-OB, S-Fe, and S-BC. The types of agricultural residue management being studied include open burning (OB), fertilizer production (Fe), and biochar production (BC) using slow pyrolysis. The three crop residues are corn residue (C); rice straw (R); and sugarcane leaves (S). Specifically, the nine agricultural residue management schemes are analyzed to determine the GWP (i.e., environmental assessment) and net cash flow (economic assessment); and the most preferable type of agricultural residue management is selected. The selection criteria are the lowest GWP impact and the highest net cash flow. The environmental assessment shows that, except the open burning schemes (C-OB, R-OB, and S-OB), fossil fuel consumption is the main contributor of the GWP impact. Meanwhile, the biochar schemes (C-BC, R-BC and S-BC) have the lowest GWP impact. The economic assessment outcomes show that the biochar schemes have the highest net cash flow, with the biochar production from corn residue (C-BC) generating the highest cash inflow. The revenue from the sale of biochar is highest, followed by the sale of bio-oil. The expenditures of the open burning schemes are practically zero, while the biochar schemes are the most costly to operate. The most preferable agricultural residue management type is the biochar production (C-BC, R-BC and S-BC), given the lowest GWP impact and the highest net cash flow. As a result, the more eco-friendly biochar schemes should be adopted as an alternative to open burning of crop residues. To discourage open burning and promote the adoption of zero burning practices, especially the biochar production, the authorities need to tailor the government assistance programs to the requirements of farmers and make them more accessible to the farmers. The government assistance programs could be in the form of, e.g., zero-interest loans and a zero rate of import customs duty on land clearing machines and equipment.

## Data Availability

The datasets generated and/or analyzed during the current study are available in this article.
